# Traceability analysis of a foodborne disease outbreak caused by *Clostridium perfringens* in Ma’anshan City, Anhui Province, 2024

**DOI:** 10.3389/fpubh.2025.1683405

**Published:** 2026-01-05

**Authors:** Li Wang, Ying Luo, Yuanting Sun, Fen Lu, Yiqun Chen, Liangliang Jiang

**Affiliations:** 1 Department of Microbiology and Laboratory Medicine, Ma'anshan Center for Disease Control and Prevention (Ma'anshan Health Supervision Institute), Ma'anshan, China; 2 School of Public Health, Anhui Medical University, Hefei, China

**Keywords:** *Clostridium perfringens*, foodborne diseases, toxin gene, whole-genome sequencing, traceability analysis

## Abstract

**Objectives:**

This study aimed to perform laboratory testing and traceability analysis of suspicious samples from a foodborne disease outbreak caused by *Clostridium perfringens* (*C. perfringens*) in a hospital in Ma’anshan City, Anhui Province, and to evaluate the application of whole-genome sequencing technology in the tracing of foodborne disease outbreaks.

**Methods:**

On-site epidemiological investigations were conducted, and suspicious biological, food, and environmental samples were collected. Patient biological samples underwent multi-pathogen screening. Following the epidemiological analysis, isolation, culture, and identification of suspected pathogens were carried out. Toxin gene detection and whole-genome sequencing were then performed on the isolated *C. perfringens* strains.

**Results:**

A total of 131 samples were collected during the outbreak. Among these, 20 anal swabs from patients with diarrhoea were tested for the nucleic acids of foodborne pathogens and five types of Diarrheagenic *Escherichia coli*. All samples tested positive for *C. perfringens* nucleic acid, while six samples also tested positive for enteropathogenic *E. coli*. Isolation and culture revealed that *C. perfringens* was detected in 41 samples, with a detection rate of 31.3% (41/131). Nucleic acid detection of six toxin genes (*plc*, *cpb*, *cpe*, *Ia*, *etx*, and *netB*) was performed on the 41 *C. perfringens* strains, with 29 strains testing positive for *cpe*. Whole-genome sequencing of the *cpe*-positive strains revealed identical ST sequence types and a maximum of 25 SNP differences, indicating a highly clonal group. Phylogenetic analysis of the core genome demonstrated homology among *C. perfringens* strains from patient anal swabs, canteen employees’s anal swabs, and food samples.

**Conclusion:**

The outbreak was likely caused by contamination of Chinese food by *C. perfringens* from infected canteen employees. Whole-genome sequencing was instrumental in accurately tracing the source of the outbreak.

## 1 Introduction

*Clostridium perfringens* (*C. perfringens*) is a widespread bacterium commonly associated with food poisoning and is prevalent in the natural environment, particularly in animal intestines (1). It produces a variety of toxins, with enterotoxin being the primary cause of foodborne illness. Alpha toxin, a key enterotoxin, disrupts the junctions between intestinal cells, resulting in cell death. Additionally, certain strains produce CPE toxin, a critical factor in food poisoning and non-foodborne diarrhoea (2, 3). *C. perfringens*-related foodborne outbreaks are frequent worldwide, often involving dozens or even hundreds of cases, posing significant social and healthcare challenges. In the United States, *C. perfringens* ranks as the second most common foodborne pathogen (4), responsible for approximately 1 million illnesses annually. In the United Kingdom, it is the third most common cause of bacterial foodborne disease in England and Wales (5). In China, while there have been a number of reports on *C. perfringens*-induced, the full spectrum of its epidemiological characteristics and disease impact remains to be fully elucidated. This is largely due to constraints in cultivation conditions and detection techniques in some settings, which may still lead to an underestimation of its actual disease burden in specific regions or populations. In this outbreak, multi-pathogen screening facilitated rapid identification of the pathogen (6). For the isolated positive strains, fluorescent Polymerase Chain Reaction (PCR) was employed to detect toxin genes (7), and whole-genome sequencing was used for source tracing through Multilocus Sequence Typing (MLST), Single-nucleotide polymorphism (SNP) analysis, and the construction of a core genome phylogenetic tree (8). This investigation confirmed that the foodborne illness was caused by type F *C. perfringens*, marking the first reported *C. perfringens* foodborne outbreak in Anhui Province in recent years.

## 2 Methods

### 2.1 Basic information of the incident

Between the evening of August 6th and the early morning of August 7th, 2024, multiple cases of diarrhoea occurred among patients in a specific inpatient ward at a hospital in Ma’anshan City. Upon receiving the report, the local disease control centre promptly initiated an epidemiological investigation on the morning of August 7th. On August 6th, the ward had 93 inpatients and 7 medical staff. Lunch was served at 11:00 a.m., with most individuals consuming meals prepared by the hospital cafeteria. Among them, 18 patients and 2 medical staff requested additional servings of roast duck, which was sourced from an external restaurant and cut and served by the cafeteria staff. There are 8 hospital canteen employees (A–H), among whom A is responsible for cutting and preparing, B for meal delivery, C and H for meal selling, D for dishwashing, E and F for cooking, and G is the head chef. The roast duck was purchased by canteen employee E from an off-campus roast duck shop at 8:00 on August 6. After bringing it to the canteen, A was responsible for cutting and plating the roast duck, H for portioning other dishes to be sent to the inpatient ward, and finally B for delivering them to a certain inpatient ward. Between 19:00 on August 6th and 01:00 on August 7th, all 20 individuals who consumed the roast duck developed symptoms of diarrhoea and abdominal pain, but none had a fever. The incubation period ranged from 8 to 14 h, with a median of 12 h. Those who did not consume the roast duck during lunch on August 6th did not experience illness.

### 2.2 Sample collection

A total of 131 samples were collected during the outbreak investigation, including 72 anal swabs (8 from canteen employees, 2 from roast duck shop employees, 20 from diarrhoea cases, and 42 from co-exposed individuals), 10 hand smear samples (8 from canteen employees, 2 from roast duck shop employees), 17 environmental swabs from the canteen, 7 environmental swabs from the roast duck shop, 14 food samples from the canteen, 2 food samples from the roast duck shop, and 9 water samples from the canteen’s pipeline network. All samples were transported under low temperature conditions to the Ma’anshan Center for Disease Control and Prevention laboratory. Nucleic acid multiplex screening for foodborne pathogens and five types of diarrhoea-causing *Escherichia coli* was initially conducted on the 20 anal swab samples from diarrhoea cases. Concurrently, pathogen isolation and culture were performed on all the samples.

### 2.3 Laboratory preliminary screening tests

Twenty anal swab samples from the reported diarrhoea cases were selected for analysis. Nucleic acids were extracted using an automatic nucleic acid extractor. Multiplex nucleic acid screening for foodborne pathogenic bacteria (The pathogens include: *Staphylococcus aureus*, *Salmonella* spp., *Escherichia coli* O157, *Listeria monocytogenes*, *Cronobacter* spp., *Yersinia enterocolitica*, *Vibrio* spp., *Shigella* spp., *Bacillus cereus*, *Aeromonas* spp., *C. perfringens*, *Campylobacter* spp.) and nucleic acid detection for five types of diarrhoea-causing *E. coli* were performed according to the protocols of the multiplex real-time fluorescent PCR detection kit for foodborne pathogens (Product No.: A552LYH; Zhuocheng Huisheng, Beijing, China) and the nucleic acid detection kit (PCR-fluorescent probe method) for five types of Diarrheagenic *E. coli* (Product No.: A714LYH; Zhuocheng Huisheng, Beijing, China).

### 2.4 Sample culture and identification

The collected water samples were tested for total colony counts, total coliforms, and *C. perfringens*, following the detection method outlined in GB/T 5750.12-2023. Anal swab, hand smear, environmental smear, and food samples were cultured to isolate *C. perfringens* and Diarrheagenic *E. coli* strains. For the detection of *C. perfringens* in food samples, the national standard GB 4789.13–2012 was adopted: 25 g of food sample was added to 225 mL of 0.1% peptone water to prepare a 10^−1^ dilution; 1 mL of the above 10^−1^ dilution was added to 9 mL of 0.1% peptone water to prepare serial dilutions from 10^−2^ to 10^−5^. 1 mL of each dilution was pipetted into sterile Petri dishes (two dishes per dilution), followed by pouring Tryptose-Sulfite-Cycloserine (TSC) Agar Base (Luqiao, Beijing, China). The dishes were anaerobically incubated at 36 °C for 24 h for colony counting. For other samples, after homogenization, they were directly streaked onto *C. perfringens* Chromogenic Agar Plates (Chromagar, Paris, France). Nucleic acids of suspected strains isolated from all samples were extracted by boiling method and then identified using the *C. perfringens* Nucleic Acid Real-Time Fluorescent PCR Detection Kit (Product No.: FXJ024Y-50; Xinzhong Biology, Wuhan, China). For the detection of Diarrheagenic *E. coli* in food samples, the national standard GB 4789.6-2016 was followed: 25 g of food sample was added to 225 mL of Enteric Enrichment Broth (Luqiao, Beijing, China), homogenized, and incubated at 42 °C for 18 h, then streaked onto MacConkey Agar (MAC) Plates (Luqiao, Beijing, China). For other samples, after homogenization, they were directly streaked onto MAC plates. Nucleic acids of suspected strains isolated from all samples were extracted by boiling method and then typed and identified using the nucleic acid detection kit (PCR-fluorescent probe method) for five types of Diarrheagenic *E. coli* (Product No.: A714LYH; Zhuocheng Huisheng, Beijing, China).

### 2.5 Toxin gene detection

The *C. perfringens* strains isolated through cultivation had their nucleic acids extracted using a bacterial Deoxyribonucleic Acid (DNA) extraction kit (Tianlong, Xi^,^an, China). Using fluorescent PCR, six toxin genes (*plc*, *cpb*, *cpe*, *Ia*, *etx*, and *netB*) were detected with the *C. perfringens* toxin gene nucleic acid detection kit (Product No.: IDL023Y-25; Xinzhong Biology, Wuhan, China). Based on the results of the toxin gene detection, the *C. perfringens* strains were classified into pathogenic types (Table 1) (9, 10).

**Table 1:** Virulence analysis of *C. perfringens* and related diseases.

Strain type	The main toxin types produced						Related diseases	
	*cpa/plc* (α)	*cpb* (β)	*etx* (ε)	*Ia* (ι)	*cpe* (CPE)	*netB* (NetB)	Person	Animal
A	+	−	−	−	−	−	Gas gangrene	Gas gangrene; hemorrhagic enteritis
B	+	+	+	−	−	−		Necrotic enteritis in cattle and horses, etc.
C	+	+	−	−	±	−	Acute hemorrhagic necrotic enteritis	Necrotic enteritis of newborn animals
D	+	−	+	−	±	−		Enterotoxemia in sheep and other animals
E	+	−	−	+	±	−		It may be related to enteritis in cattle and rabbits.
F	+	−	−	−	+	−	Food poisoning; sporadic diarrhoea, etc.	
G	+	−	−	−	−	+		Necrotic enteritis in poultry

“+” indicates the presence of a specific virulence gene or toxin; “±” indicates that the strain may or may not carry a specific virulence gene or toxin; The blank indicates that this type of strain has not been identified as capable of causing disease in humans or animals. The α, β, ε, ι, CPE, and NetB toxins are encoded by the *cpa/plc*, *cpb*, *etx*, *iap*, *cpe*, and *netB* genes, respectively.

### 2.6 Whole-genome sequencing, MLST typing, SNP analysis, and construction of the core genome phylogenetic tree

Whole-genome sequencing of the genomic DNA from cultivated *C. perfringens* and enteropathogenic *E. coli* was conducted using the MGISEQ-200 platform from BGI (Beijing Genomics Institute). The reagents employed included the MGIEasy Fast Restriction Enzyme Library Preparation Kit (Product No.: 940-000027-00; MGI Tech, Wuhan, China), the DNBSEQ One-Step DNB Preparation Kit (Product No.: 1000026466; MGI Tech, Wuhan, China), and the MGISEQ-200RS High-Throughput Sequencing Kit (Product No.: 1000019844; MGI Tech, Wuhan, China).

Microobench was utilized for comprehensive analysis of the genomic sequencing data from 41 *C. perfringens* strains. Quality control of the raw Next-Generation Sequencing (NGS) sequencing data was conducted through FastQC v0.11.5 (11) and Trimmomatic v0.36 (12). The assembly process was carried out using SPAdes v3.13.0, and checkM was employed to assess the quality of the assembled genomes (13). MLST analysis was performed on the assembled genomes using mlst v2.23.0 (14) and chewBBACA v2.8.5 (15), referencing the PubMLST (16) and gcPathogen databases (17). For SNP analysis, the whole genome of *C. perfringens* strain 24MASCDC4662, isolated from the anal swab of a cafeteria food seller, was used as the reference sequence. Snippy software (18) was used to perform SNP analysis on the genomic data of the 41 *C. perfringens* strains. The allelic results from the core genes of the 41 strains were compiled to generate the allelic genotype matrix for these core genes. This matrix was then input into ReporTree software (19), and hierarchical clustering analysis was performed using the single-linkage algorithm, producing genetic clustering results based on the core genotypes.

## 3 Results

### 3.1 Results of the initial sample screening

A multiplex nucleic acid screening for foodborne pathogenic bacteria and a nucleic acid screening for five types of Diarrheagenic *E. coli* were conducted on the anal swabs from 20 diarrhoea cases. The results revealed that all 20 samples were positive for *C. perfringens*, and 6 samples were tested positive for enteropathogenic *E. coli*, with the virulence gene *eae* present in all of these positive samples. Additionally, 1 sample tested positive for *Bacillus cereus*, 1 for *Cronobacter sakazakii*, and the test results for other pathogens were negative.

### 3.2 Results of the sample isolation and culture

The total number of colonies and total coliforms were analyzed in 9 water samples. The results showed that both Dishwashing Room Water Sample 1 and Stove Pool Water Sample 2 had total colony counts exceeding 100 CFU/mL. The lactose fermentation tests for Dishwashing Room Water Sample 2 and Cutting and Preparation Room Water Sample 3 were both positive. A total of 131 samples were cultured for *C. perfringens*, yielding 41 positive samples, corresponding to a detection rate of 31.3% (41/131). The 41 *C. perfringens* strains were isolated from 19 anal swabs of diarrhoea cases, 5 anal swabs of canteen employees, 2 food reservation samples from the canteen (beef dices and cauliflower from the lunch on August 6th,the quantitative results were 1.3 × 10^4^ CFU/g and 8.6 × 10^3^ CFU/g, respectively), 14 anal swabs from co-exposed individuals, and 1 anal swab from an employee at the roast duck shop. *C. perfringens* was not detected in the roast duck sample. Enteropathogenic *E. coli* was detected in 3 samples, with all virulence genes testing positive for *eae*. These samples included one anal swab from a canteen employee and two anal swabs from diarrhoea cases (Table 2).

**Table 2:** Sample detection results.

Sample type	Sample source	Number of samples	PCR preliminary screening		Isolation and culture	
			Detection items	Number of positive nucleic acid samples	Detection items	Number of detected samples
Anal swab	Diarrhoea cases	20	Multiplex nucleic acid detection of foodborne pathogenic bacteria	20	*C. perfringens*	19
			Nucleic acid detection of five types of Diarrheagenic *Escherichia coli*	6	Diarrheagenic *Escherichia coli*	2
	Employees of the roast duck shop	2	/	/	*C. perfringens*	1
			/	/	Diarrheagenic *Escherichia coli*	0
	Canteen employees	8	/	/	*C. perfringens*	5
			/	/	Diarrheagenic *Escherichia coli*	1
	Co-exposed individuals	42	/	/	*C. perfringens*	14
			/	/	Diarrheagenic *Escherichia coli*	0
Hand smear sample	Canteen employees	8	/	/	*C. perfringens*	0
			/	/	Diarrheagenic *Escherichia coli*	0
	Employees of the roast duck shop	2	/	/	*C. perfringens*	0
			/	/	Diarrheagenic *Escherichia coli*	0
Environmental smear sample	Canteen	17	/	/	*C. perfringens*	0
			/	/	Diarrheagenic *Escherichia coli*	0
	Roast duck shop	7	/	/	*C. perfringens*	0
			/	/	Diarrheagenic *Escherichia coli*	0
Food sample	Reserved food samples from the canteen	14	/	/	*C. perfringens*	2
			/	/	Diarrheagenic *Escherichia coli*	0
	Food samples from the roast duck shop	2	/	/	*C. perfringens*	0
			/	/	Diarrheagenic *Escherichia coli*	0
Water sample	Water samples from the canteen pipeline network	9	/	/	*C. perfringens*	0
Total		131	/	/	/	/

“/” indicates that the item was not tested.

“Co-exposed individuals” refers to individuals who consumed meals distributed by the hospital cafeteria in the same ward but did not eat the roast duck.

### 3.3 Results of toxin gene detection and pathogenic type classification

Nucleic acids were extracted from 41 isolated *C. perfringens* strains, and fluorescent PCR was performed to detect 6 toxin genes. The results indicated that *cpb*, *etx*, *Ia*, and *netB* were negative in all strains, while *plc* was positive in all. The *cpe* gene was detected in 29 strains, which included 19 anal swabs from diarrhoea cases, 4 anal swabs from canteen employees, 2 food reservation samples from the canteen, and 4 anal swabs from co-exposed individuals. The remaining 12 strains were negative for *cpe* (Table 3). Among them, the 4 canteen staff members who were detected with *cpe*-positive *C. perfringens* are A (catering staff), B (food delivery staff), E (chef), and H (food server). Based on the toxin gene detection, the 29 *cpe*-positive strains were classified as type F, while the 12 *cpe*-negative strains were identified as type A.

**Table 3:** Detection results of toxin genes in 41 strains of *C. perfringens.*

Strain number	Strain source	Toxin genes					
		*plc*	*cpb*	*netB*	*etx*	*Ia*	*cpe*
24MASCDC4622	Anorectal swab for diarrhoea cases	+	−	−	−	−	+
24MASCDC4623		+	−	−	−	−	+
24MASCDC4624		+	−	−	−	−	+
24MASCDC4625		+	−	−	−	−	+
24MASCDC4626		+	−	−	−	−	+
24MASCDC4627		+	−	−	−	−	+
24MASCDC4628		+	−	−	−	−	+
24MASCDC4629		+	−	−	−	−	+
24MASCDC4630		+	−	−	−	−	+
24MASCDC4631		+	−	−	−	−	+
24MASCDC4632		+	−	−	−	−	+
24MASCDC4633		+	−	−	−	−	+
24MASCDC4634		+	−	−	−	−	+
24MASCDC4635		+	−	−	−	−	+
24MASCDC4636		+	−	−	−	−	+
24MASCDC4637		+	−	−	−	−	+
24MASCDC4638		+	−	−	−	−	+
24MASCDC4639		+	−	−	−	−	+
24MASCDC4640		+	−	−	−	−	+
24MASCDC4648	Anorectal swab of canteen employees	+	−	−	−	−	+
24MASCDC4650		+	−	−	−	−	+
24MASCDC4652		+	−	−	−	−	−
24MASCDC4656		+	−	−	−	−	+
24MASCDC4662		+	−	−	−	−	+
24MASCDC4705	Food reserved samples from the canteen	+	−	−	−	−	+
24MASCDC4708		+	−	−	−	−	+
24MASCDC4680	Anorectal swab from employees of the roast duck shop	+	−	−	−	−	−
24MASCDC4681	Anorectal swab of co-exposed individuals	+	−	−	−	−	+
24MASCDC4692		+	−	−	−	−	+
24MASCDC4693		+	−	−	−	−	+
24MASCDC4694		+	−	−	−	−	−
24MASCDC4697		+	−	−	−	−	+
24MASCDC4699		+	−	−	−	−	−
24MASCDC4700		+	−	−	−	−	−
24MASCDC4715		+	−	−	−	−	−
24MASCDC4717		+	−	−	−	−	−
24MASCDC4721		+	−	−	−	−	−
24MASCDC4722		+	−	−	−	−	−
24MASCDC4724		+	−	−	−	−	−
24MASCDC4726		+	−	−	−	−	−
24MASCDC4728		+	−	−	−	−	−

### 3.4 Whole-genome sequencing analysis results

#### 3.4.1 Basic genomic information of 41 strains of *C. perfringens*

The whole-genome sequencing data of the 41 *C. perfringens* strains were assembled, and the quality of the assemblies was assessed using checkM. The quality values (Q30) ranged from 91.6 to 94.5%, all exceeding 90%. The G+C content of the original sequences ranged from 28.6 to 34.6%, with the G+C content of the 29 *cpe*-positive strains falling between 28.6% and 29.6%. The read mapping rate was greater than 98.0%, and the coverage rate at 30× was above 98.0%.

#### 3.4.2 MLST typing analysis

MLST analysis was conducted on the assembled sequences using mlst v2.23.0. Among the 41 *C. perfringens* strains, 8 strains were identified as known types, namely ST210 (2 strains), ST324, ST326, ST396, ST452, ST649, and ST670. The remaining 33 strains were of unknown types, which could be divided into 3 distinct ST types based on the characteristics of allelic gene coding. No matching registered ST type serial numbers were found after comparison with the PubMLST database, indicating that these 3 ST types are novel sequence types. Subsequently, the relevant strain information, genomic data and assembled sequences were submitted to the PubMLST database, and officially named as ST1156, ST1157 and ST1158 after review (Table 4). Notably, among the 29 *cpe*-positive *C. perfringens* strains, the allelic gene numbers of all strains were completely consistent, specifically *colA* (23), *groEL* (12), *sodA* (24), *plc* (20), *gyrB* (18), *sigK* (17), *pgk* (14) and *nadA* (20), suggesting that these 29 *cpe*-positive strains belong to the same ST sequence type (ST1156).

**Table 4:** Genotyping characteristics of *C. perfringens.*

Strain number	Strain source	MLST									Number of differences in SNP loci	Biotype
		*colA*	*groEL*	*sodA*	*plc*	*gyrB*	*sigK*	*pgk*	*nadA*	ST		
24MASCDC4622	Anorectal swab for diarrhoea cases	23	12	24	20	18	17	14	20	ST1156	20	Type F
24MASCDC4623		23	12	24	20	18	17	14	20	ST1156	25	Type F
24MASCDC4624		23	12	24	20	18	17	14	20	ST1156	16	Type F
24MASCDC4625		23	12	24	20	18	17	14	20	ST1156	11	Type F
24MASCDC4626		23	12	24	20	18	17	14	20	ST1156	10	Type F
24MASCDC4627		23	12	24	20	18	17	14	20	ST1156	6	Type F
24MASCDC4628		23	12	24	20	18	17	14	20	ST1156	6	Type F
24MASCDC4629		23	12	24	20	18	17	14	20	ST1156	6	Type F
24MASCDC4630		23	12	24	20	18	17	14	20	ST1156	19	Type F
24MASCDC4631		23	12	24	20	18	17	14	20	ST1156	20	Type F
24MASCDC4632		23	12	24	20	18	17	14	20	ST1156	5	Type F
24MASCDC4633		23	12	24	20	18	17	14	20	ST1156	4	Type F
24MASCDC4634		23	12	24	20	18	17	14	20	ST1156	5	Type F
24MASCDC4635		23	12	24	20	18	17	14	20	ST1156	4	Type F
24MASCDC4636		23	12	24	20	18	17	14	20	ST1156	4	Type F
24MASCDC4638		23	12	24	20	18	17	14	20	ST1156	4	Type F
24MASCDC4639		23	12	24	20	18	17	14	20	ST1156	9	Type F
24MASCDC4640		23	12	24	20	18	17	14	20	ST1156	7	Type F
24MASCDC4641		23	12	24	20	18	17	14	20	ST1156	6	Type F
24MASCDC4648	Anorectal swab of canteen employees	23	12	24	20	18	17	14	20	ST1156	3	Type F
24MASCDC4650		23	12	24	20	18	17	14	20	ST1156	7	Type F
24MASCDC4652		112	34	1	130	4	4	3	1	ST396	54,517	Type A
24MASCDC4656		23	12	24	20	18	17	14	20	ST1156	8	Type F
24MASCDC4662		23	12	24	20	18	17	14	20	ST1156	0	Type F
24MASCDC4705	Food reserved samples from the canteen	23	12	24	20	18	17	14	20	ST1156	2	Type F
24MASCDC4708		23	12	24	20	18	17	14	20	ST1156	1	Type F
24MASCDC4680	Anorectal swab from employees of the roast duck shop	5	4	102	24	38	61	8	105	ST1157	54,076	Type A
24MASCDC4681	Anorectal swab of co-exposed individuals	23	12	24	20	18	17	14	20	ST1156	3	Type F
24MASCDC4692		23	12	24	20	18	17	14	20	ST1156	1	Type F
24MASCDC4693		23	12	24	20	18	17	14	20	ST1156	2	Type F
24MASCDC4694		19	130	1	5	5	2	2	3	ST452	49,760	Type A
24MASCDC4697		23	12	24	20	18	17	14	20	ST1156	4	Type F
24MASCDC4699		29	19	159	3	3	5	1	13	ST649	51,513	Type A
24MASCDC4700		4	19	1	4	3	5	1	1	ST326	50,691	Type A
24MASCDC4715		6	101	93	5	5	2	2	3	ST210	49,346	Type A
24MASCDC4717		1	12	18	184	16	96	44	153	ST1158	22,911	Type A
24MASCDC4721		1	12	18	184	16	96	44	153	ST1158	22,852	Type A
24MASCDC4722		1	12	18	184	16	96	44	153	ST1158	22,828	Type A
24MASCDC4724		6	101	93	5	5	2	2	3	ST210	47,317	Type A
24MASCDC4726		19	153	1	5	5	2	3	1	ST670	46,534	Type A
24MASCDC4728		3	29	3	10	3	5	20	1	ST324	47,653	Type A

After assembling and splicing the whole-genome sequencing data of 3 strains of enteropathogenic *E. coli*, the analysis revealed that 2 strains, sourced from the anorectal swabs of diarrhoea cases, were of the same ST type, ST20, while the isolate from the anorectal swab of a canteen employee was classified as ST6272.

#### 3.4.3 SNP analysis

For SNP analysis of the 41 *C. perfringens* strains, the whole genome of *C. perfringens* strain 24MASCDC4662, isolated from the anorectal swab of a canteen food seller, was used as the reference sequence. The results indicated that among the 29 *C. perfringens* strains positive for *cpe*, the number of SNP site differences was ≤ 25, indicating that these 29 strains had a highly consistent genetic background and belonged to the same highly clonal bacterial population. In contrast, the number of SNP differences between these 29 strains and the other 12 *cpe*-negative *C. perfringens* isolates ranged from 22,828 to 54,517, suggesting that the two types of strains had no close genetic relationship and belonged to different genetic branches. Meanwhile, it was found that the number of SNP site differences between the *C. perfringens* strains detected in 2 food samples from the canteen’s reserved samples and the reference strain (the strain isolated from the anorectal swab of a canteen food seller) was <3, indicating that the strains detected in the food samples were highly homologous to the strains carried by the canteen food seller.

#### 3.4.4 Construction of the core genome phylogenetic tree

**Figure 1:**
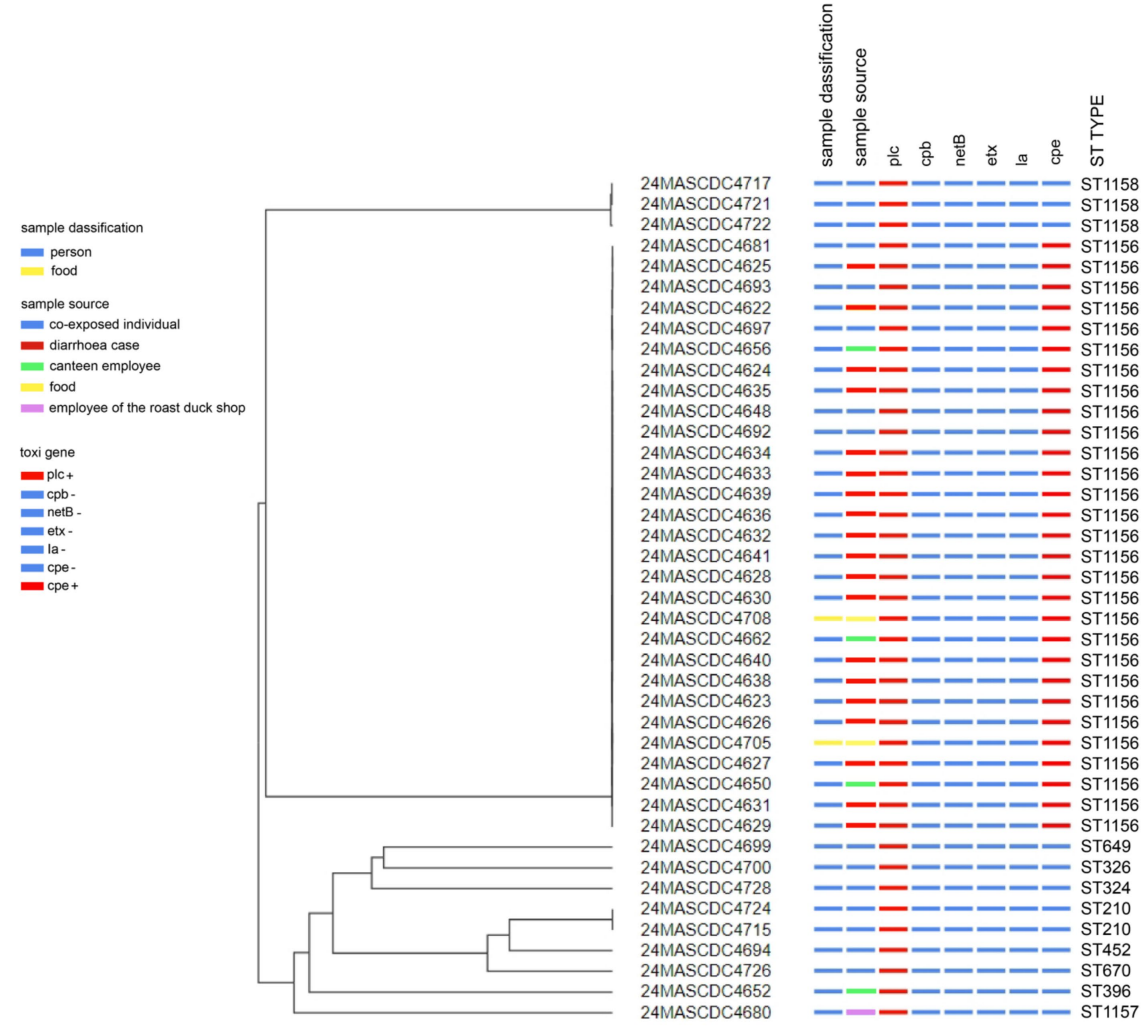
Phylogenetic tree of the core genome of 41 strains of *C. perfringens.*

A core genome phylogenetic tree was constructed based on the genomes of the 41 *C. perfringens* strains. It can be seen that the core genomes of the 29 *cpe*-positive *C. perfringens* strains were completely clustered together to form a single clade, indicating a high consistency in their origins. The 29 *cpe*-positive *C. perfringens* strains and the remaining 12 *cpe*-negative *C. perfringens* strains belonged to completely different clades on the phylogenetic tree. The clades of the two types of strains were far apart in the tree, with no cross-clustering phenomenon observed (Figure 1).

## 4 Discussion

Food poisoning incidents caused by *C. perfringens* have been widely reported, particularly in collective catering settings such as hospitals, school cafeterias, prisons, and nursing homes (20). In the United States, *C. perfringens* is responsible for an estimated 1 million cases of foodborne illnesses annually. In recent years, food poisoning outbreaks attributed to *C. perfringens* have been reported in Beijing, Zhengzhou, Suzhou, and Guizhou Province, China (20–23). The epidemiological investigation of this incident revealed that all 20 cases had consumed the same food (roast duck) during the same meal, exhibiting a point-source exposure pattern with no secondary cases. The incubation period ranged from 8 to 14 h, with a median of 12 h. The primary clinical symptoms were diarrhoea and abdominal pain, without fever, consistent with the typical clinical presentation of foodborne illnesses caused by *C. perfringens* (24). These findings suggest that this is a suspected foodborne disease outbreak.

The roast duck involved in the incident was purchased from an external vendor by the hospital canteen. However, both the epidemiological investigation and laboratory tests indicated that the roast duck was not contaminated with *C. perfringens* prior to its arrival at the canteen. First, the reserved sample of the roast duck, collected before it was distributed to the inpatient ward at noon, was negative for *C. perfringens*. Second, although *C. perfringens* was detected in the anorectal swab of a roast duck shop employee, the *cpe* toxin gene was absent, suggesting that the *C. perfringens* detected in the employee’s swab was not the same strain responsible for the outbreak. In contrast, *C. perfringens* was detected in the anorectal swabs of multiple diarrhoea cases, canteen employees, and the reserved food samples, with toxin gene profiles showing consistency. This evidence strongly supports the conclusion that the outbreak was caused by *C. perfringens* contamination from infected canteen employees, who then contaminated the food. Among those exposed to the same meal in the ward, some individuals infected with *C. perfringens* may not have developed symptoms or experienced only mild symptoms due to a low exposure dose. A canteen catering staff member responsible for cutting and portioning roast duck was found to have *C. perfringens* isolated from their anal swab specimen genetically identical to that from the case’s anal swab. This suggests that the catering staff member, who carried *C. perfringens*, contaminated food—particularly roast duck—during the meal distribution and sale process, leading to illness in multiple diners. This incident underscores the importance of enhancing food safety awareness among canteen employees, as well as adhering to standardized self-health monitoring practices. Canteen managers should also strengthen food safety training to ensure that staff strictly follow food safety protocols, effectively preventing the clustering and outbreak of foodborne diseases.

The pathogenicity of *C. perfringens* is attributed to the toxins it produces (25). Currently, the main toxins studied include four exotoxins—*α*, *β*, *ε*, *ι*—along with other toxins such as CPE, NetB, PFO, and TpeL. Based on the production of different toxins, *C. perfringens* is classified into seven types: A, B, C, D, E, F, and G (9, 26), with types A, C, and F being primarily responsible for human diseases. Among these, type F *C. perfringens*, which produces the enterotoxin CPE and is positive for either *cpa* or *plc*, is the most commonly reported type causing food poisoning worldwide (27, 28). The 41 *C. perfringens* strains detected in this outbreak all carried the *plc* toxin gene, and 29 strains were simultaneously positive for the *cpe* toxin gene, aligning with findings from previous reports.

With the rapid advancement of modern molecular biology techniques, tools such as real-time fluorescence quantitative PCR, multiplex nucleic acid screening, and whole-genome sequencing have become integral in handling and tracing foodborne outbreaks. Multi-pathogen nucleic acid screening technology allows for the detection of multiple potential pathogens within a short timeframe and offers high sensitivity and specificity, making it crucial for the swift response and management of food poisoning incidents. Compared to traditional methods like animal toxin neutralization tests and toxin antibody detection kits, molecular detection techniques enable quicker, more sensitive, and cost-effective identification of toxin genes, which aids in determining the pathogen types and provides significant guidance for clinical diagnosis and treatment (29). With the continuous evolution and mutation of bacteria, new pathogenic strains are constantly emerging. MLST typing analysis allows for the rapid identification of these new strains by comparing them to known reference strains, enabling the determination of their taxonomic status and genetic characteristics. This is crucial for the timely identification and response to emerging pathogen threats (30). SNP analysis plays a pivotal role in foodborne disease tracing, providing essential information to pinpoint the cause of the outbreak, as well as tracking the source of contamination and the transmission route. By conducting SNP analysis on strains isolated from different samples, it is possible to determine whether these strains share the same or similar genetic features (31). The construction of a core genome phylogenetic tree effectively illustrates the evolutionary relationships and genetic distances between strains, facilitating the determination of whether strains from different sources are homologous (32).

In this incident, multiplex pathogen nucleic acid screening was employed as the first step to swiftly identify *C. perfringens* as the suspected pathogen, guiding the subsequent investigation. Next, PCR was used to detect toxin genes in the *C. perfringens* strains obtained through cultivation. The results indicated that 29 out of 41 strains carried consistent toxin genes (*plc*+, *cpe*+), confirming that the pathogen responsible for the outbreak was type F *C. perfringens* producing enterotoxin CPE. Finally, whole-genome sequencing was performed on the 41 *C. perfringens* strains, and through MLST typing analysis, SNP analysis, and the construction of a core genome phylogenetic tree, it was determined that the *C. perfringens* strains isolated from the anorectal swabs of diarrhoea cases, food reserve samples, and the anorectal swabs of canteen employees all originated from the same source, accurately tracing the cause of the foodborne outbreak. The number of SNP site differences between the *C. perfringens* strains detected in 2 food samples from the canteen’s reserved samples and the strain isolated from the food seller’s anal swab (strain number: 24MASCDC4662) was < 3. It is speculated that the *cpe*-positive *C. perfringens* strains detected in the food samples in this incident were most likely derived from the strains carried by the canteen food seller. As a carrier of *cpe*-positive *C. perfringens*, the food seller may have transmitted the highly clonal *cpe*-positive strains carried by themselves to the food through hand contact, operational contamination and other ways during food processing and sales, ultimately leading to the detection of strains highly homologous to the strain isolated from the food seller’s anal swab in the food samples.

## 5 Conclusion

Through epidemiological investigation and multi-dimensional laboratory testing, this study identified that the foodborne disease outbreak in Ma’anshan City was caused by enterotoxin (CPE)-producing *C. perfringens* type F. The transmission chain involved canteen staff carrying the pathogenic bacteria, who contaminated food during the process of cutting and selling roast duck, ultimately leading to illness in multiple individuals. This incident marks the first reported case of such a *C. perfringens* foodborne outbreak in Anhui Province. It not only fills the gap in epidemiological data on this type of disease in the region but also provides three key references for subsequent foodborne disease prevention and control efforts in the area: (1) It clarifies that collective catering establishments (especially hospital canteens) need to focus on real-time health monitoring of employees and strengthen screening for pathogenic bacteria carriage; (2) It verifies the core role of molecular technologies such as multiplex nucleic acid screening and whole-genome sequencing (including MLST typing and SNP analysis) in the rapid traceability of foodborne diseases, providing a technical paradigm for the efficient handling of similar incidents; (3) It reminds regulatory authorities to further enhance food safety training for collective catering units, promote the implementation of operational standards, and reduce the risk of foodborne disease outbreaks at the source.

## Data Availability

The datasets presented in this study can be found in online repositories. The names of the repository/repositories and accession number(s) can be found in the article/supplementary material.
